# Optimising the PTSD Hub App Through Co‐Production: Enhancing Digital Support for PTSD Management in Primary Care

**DOI:** 10.1111/hex.70598

**Published:** 2026-02-08

**Authors:** Natasha Tyler, Sarah Croke, Chloe‐Nicole Low, Nicola Cassidy, Evgenia Gkintoni, Brian McMillan, Maria Panagioti

**Affiliations:** ^1^ NIHR School for Primary Care Research, Division of Population Health, Health Services Research and Primary Care The University of Manchester Manchester UK; ^2^ School of Medicine University of Nottingham Nottingham UK; ^3^ PTSD Hub Hyde England UK; ^4^ Lab of Public Health, Epidemiology & Quality of Life, Department of Medicine University of Patras Patras Greece; ^5^ Department of Psychiatry University General Hospital of Patras Rio Greece

**Keywords:** co‐creative research, digital interventions, nominal group discussion technique, peer support, posttraumatic stress disorder, shared decision‐making

## Abstract

**Background:**

Post‐traumatic stress disorder (PTSD) affects approximately 1 in 20 individuals, with primary care playing a crucial role in early identification and management. However, PTSD is frequently underdiagnosed in primary care settings. The COVID‐19 pandemic has exacerbated the incidence of PTSD, leading to an increased demand for accessible mental health services. While NICE guidelines recommend trauma‐focused therapies, access remains limited, underscoring the need for alternative strategies to support management within primary care.

**Aim:**

To optimise and refine through co‐production the PTSD Hub app, an existing digital resource, to better support individuals with PTSD in primary care settings, improving usability, clinician engagement, and patient outcomes.

**Design & Setting:**

A co‐production approach was employed with stakeholders, including people with lived experience of PTSD, informal carers, and primary care professionals. Workshops were held online to gather insights on how to enhance the PTSD Hub app for better integration into primary care.

**Method:**

Two Nominal Group Technique workshops were conducted to identify key barriers to PTSD care in primary care and generate solutions for improving the app. Participants ranked and prioritised features based on relevance to primary care.

**Results:**

Twenty‐six participants attended the workshops, representing a diverse mix of stakeholders. Key barriers identified included stigma, clinician time constraints, and limited access to trauma‐focused therapies. Solutions included improving symptom tracking, enhancing customisation features, and raising clinician awareness.

**Conclusion:**

The study highlights the potential of the PTSD Hub app in improving and supporting PTSD management in primary care. Future research should evaluate the app's effectiveness in real‐world primary care settings.

**Patient and Public Contribution:**

Patients and members of the public were actively involved throughout this study using a co‐production approach. A lived experience advisory panel of six individuals with PTSD provided input at all stages, including shaping the study design, reviewing participant materials, and advising on workshop facilitation. Additionally, 26 participants, comprising individuals with lived experience of PTSD, informal carers, and healthcare professionals, contributed to two Nominal Group Technique workshops. A lived experience research partner (NC) was an integral part of the research team, attended all team meetings, co‐facilitated workshop sessions and contributed to design, analysis and manuscript preparation.

## 1 Introduction

Post‐traumatic stress disorder (PTSD) is a serious mental health condition that can develop after experiencing trauma. It is characterised by symptoms such as flashbacks, avoidance, negative mood changes, and heightened arousal [1]. In the UK, PTSD affects about 1 in 20 people, although the actual number may be higher due to underreporting and challenges in diagnosing the condition [2, 3]. The COVID‐19 pandemic has contributed to an increase in PTSD, as widespread trauma from illness, loss, social isolation, lockdowns, and rising rates of domestic abuse has exacerbated mental health issues [4, 5, 6]. Primary care is often the first point of contact for people with PTSD symptoms, but research shows that PTSD is often missed in primary care [7]. Barriers such as time constraints, other health conditions, and avoidance symptoms of PTSD make it harder for clinicians to diagnose and treat the condition [1]. Early treatment is important, as untreated PTSD can lead to problems like substance abuse, self‐harm, and relationship issues [8]. NICE guidelines recommend trauma‐focused therapies, such as Cognitive Behavioural Therapy, to be provided within a month of trauma, but only 24% of those who need PTSD care receive it [9, 10].

PTSD remains underrecognized and undertreated in primary care, partly due to stigma and the belief that it is untreatable [11]. This underscores the need for innovative approaches to improve PTSD care access and engagement, such as peer support and shared decision‐making. Digital peer support can reduce isolation and offer practical coping strategies by connecting individuals with others who share similar experiences [8, 12, 13]. Shared decision‐making, where patients and clinicians work together to make healthcare decisions, can improve treatment engagement and outcomes [14, 15].

Mobile applications like PTSD Hub offer a promising solution to improve patient engagement, symptom monitoring, and access to support [16]. The PTSD Hub app was developed by a person with lived experience combines evidence‐based therapies like CBT, symptom tracking, and peer support to help users manage their PTSD symptoms more effectively. A co‐author (NC) created the PTSD Hub app based on her own lived experience of navigating PTSD care. She recognised significant gaps in access to timely, trauma‐informed support and the isolation many people face when waiting for treatment. The app was developed to provide practical tools, peer support, and evidence‐based resources in a format that is accessible and user‐friendly, empowering individuals to take an active role in their recovery while bridging the gap between need and available services. The app encourages patients to actively participate in their care and recovery by integrating features like shared decision‐making.

The PTSD Hub was originally developed by one person with lived experience, drawing on her own experience of navigating PTSD care. While this foundation ensured the app addressed real‐world challenges, we recognised the need to broaden its perspective. To make the app truly inclusive and clinically relevant, the team sought input from a more diverse group of people with lived experience and healthcare professionals. Literature assessing digital interventions in mental health suggests barriers to engagement with digital solutions are often impacted by individual characteristics (i.e., gender, age, ethnicity), so gaining input from a diverse population is essential [17]. Whilst little quantitative research has tested whether endorsements from clinicians actually impact engagement rates [17], survey data suggests people indicate a greater interest in digital mental interventions that come recommended by clinicians, particularly mental health practitioners [18].

This study addresses gaps in usability and integration into primary care by involving patients, carers, and clinicians to identify barriers and propose refinements. Co‐production principles are applied to optimise an existing resource rather than create a new one, ensuring relevance and acceptability. This study explores how a stakeholder co‐produced digital mental health intervention can be optimised to better support patients with PTSD and enable its use in primary care.

## 2 Methods

### 2.1 Design

Two sequential Nominal Group Technique (NGT) workshops were conducted to refine the PTSD Hub using co‐production [19]. NGT is a structured method for group idea generation and decision‐making that encourages individual contributions and facilitates consensus [19]. NGT is a structured consensus method that ensures all participants have an equal opportunity to contribute ideas. Unlike more open group discussions method, which can be dominated by more vocal individuals, NGT incorporates silent idea generation and individual ranking, allowing quieter voices to be heard and promoting fairer decision‐making. This approach was particularly valuable for optimising the app using input from a diverse group of stakeholders, including people with lived experience and healthcare professionals, ensuring that refinements reflected a broad range of perspectives.

### 2.2 Participants

Participants were recruited via social media advertisements (X, Facebook) and email invitations from the researchers and lived experience advisory panel networks. Eligibility included individuals with lived experience of PTSD, informal carers of those with PTSD, primary care clinicians, and mental health professionals. We relied on self‐reports to confirm eligibility. Participants received compensation in the form of a shopping voucher for their time. Participants were reimbursed with a shopping voucher (£25 p/h plus +£5 digital allowance). Workshops lasted approximately 2 h each.

### 2.3 Procedure

Two online workshops were conducted to enable more people to attend at a time suitable to them, each started with an overview of the project, followed by discussions on PTSD, shared decision‐making, peer support, and findings from an evidence synthesis on digital interventions. The lived experience co‐researcher (NC) introduced the existing PTSD Hub, its purpose, and functionality. The NGT methodology was used to gather consensus on refinements, led by a researcher with experience in consensus methods (NT). Workshop 1 (8 July 2024) informed refinements to Workshop 2 (11 July 2024).

All workshops were conducted using trauma‐informed research principles to ensure participant safety and comfort. This included providing clear information about the study and consent process, allowing participants to opt out of any activity, and offering multiple ways to contribute (e.g., verbally, via chat, or anonymously). Sessions were designed to minimise distress by avoiding triggering language, including content warnings, and incorporating breaks to reduce fatigue. Researcher facilitation emphasised trust, transparency, and collaboration, with lived experience partners involved in planning and delivery. Participants were signposted to support resources after the sessions to promote wellbeing.

The workshops had four phases in line with the NGT methodology.

### 2.4 Phase 1: Silent Problem Identification

Participants individually generated lists of potential problems faced by people with PTSD, healthcare professionals supporting them, and those using the PTSD Hub app. They were given three prompts and worked with an online collaborative ‘whiteboard’ (Slido). Lists were shared, followed by a 10‐min discussion of the problems. The prompts were:

1. What problems do people living with PTSD face in the community?
2. What problems might people face using the PTSD Hub app (or other digital peer‐support solutions)?
3. What problems might GPs/clinicians face engaging with the PTSD Hub?

The prompts aimed to enable to the group to collaboratively identify problems to facilitate solutions that aim to address these problems in the context of the PTSD Hub App in Phase 2.

### 2.5 Phase 2: Silent Solution Generation

Participants independently generated lists of potential solutions, focusing on creativity without considering feasibility. They were given three prompts and asked to think broadly. Solutions were shared, followed by a 20‐min discussion to clarify and group similar ideas. The prompts were:

1. *H*ow could the PTSD Hub app be improved?
2. Which peer‐led or shared decision‐making strategies for coping with PTSD symptoms could be included in the mobile app?
3. How could we improve GP/clinician engagement with the PTSD Hub app?

### 2.6 Phase 3: Clarification

During this phase, participants shared their solutions on the same ‘whiteboard’, and a 20‐min discussion clarified and grouped similar ideas to prepare for ranking.

### 2.7 Phase 4: Ranking

Participants ranked their top three solutions based on feasibility and acceptability, producing lists for the best solutions to improve the PTSD Hub and clinician engagement. The ranking was conducted individually, followed by a group discussion. In Workshop 2, participants only ranked the best solutions after feedback from Workshop 1, simplifying the process.

### 2.8 Analysis

Each workshop produced a table of ranked solutions. Workshop 1 generated three improvement areas: acceptable improvements, feasible improvements, and best solutions for clinician engagement.

Workshop 2 focused on two improvement areas. Solutions were ranked using a simple points system (3 for first place, 2 for second, 1 for third), with totals ranked from highest to lowest. Results were presented separately for each workshop.

### 2.9 Patient and Public Involvement

A lived experience advisory panel of six individuals with PTSD provided input at all stages of the project, advising on key decisions, reviewing documents, and sharing experiences to enhance the study's delivery.

### 2.10 Ethical Approval

Ethical approval was granted by the Proportionate University of Manchester Research Ethics Committee (Ref: 2024‐19198‐34681). All participants received a written Participant Information Sheet and had the opportunity to ask questions before giving written informed consent.

## 3 Results

### 3.1 Participant Demographics

A total of 26 participants attended the two co‐production workshops, representing a broad range of stakeholders: Individuals with lived experience of PTSD, informal carers, and healthcare professionals, including primary care clinicians and secondary mental health practitioners. The sample was diverse in terms of gender, ethnicity, and geography, supporting a wide range of perspectives to inform development of the PTSD Hub, see Table 1.

**Table 1:** Participant demographics.

**Participants**					
	Stakeholder Group	Age	Gender	Ethnicity	Region of the UK
**Workshop 1**					
1	Primary care clinician	35	F	British Chinese	North West
2	Carer	59	F	White British	South East
3	Carer	56	M	British Indian	North West
4	Person Living with PTSD	58	M	White British	South East
5	Primary care clinician	33	F	White British	North West
6	Person Living with PTSD	51	M	White British	East Midlands
7	Person Living with PTSD	50	F	White British	South East
8	Primary care clinician	28	F	Mixed Race	London
9	Carer	33	N	Mixed Ethnicity	North West
10	Carer	40	F	White British	East Midlands
**Workshop 2**					
11	Primary care clinician	33	M	White British	Yorkshire
12	Primary care clinician	35	M	Chinese	North West
13	Person Living with PTSD	39	F	Mixed Ethnicity	West Midlands
14	Person Living with PTSD	26	M	Black British	London
15	Carer	30	M	Black British	Scotland
16	Primary care clinician	35	F	Asian Indian	Yorkshire
P 17	Person living with PTSD	NR	M	White British	North West
18	Carer	26	F	Black British Caribbean	South East
19	Carer	30	F	White British	Scotland
20	Person Living with PTSD	35	F	White British	Scotland
21	Carer	33	M	Mixed Ethnicity	North West
22	Person Living with PTSD	48	M	Black British‐African	North West
23	Mental Health Care Professional	37	M	Black British	North West
24	Person Living with PTSD	42	F	White British	East Midlands
25	Person living with PTSD	57	F	White British	North West
26	Carer	54	F	British Pakistani	East Midlands

*Note:* Table 1 illustrates the demographic breakdown across four categories: (1) Gender, (2) Stakeholder representation, (3) Ethnicity, and (4) Geographical distribution.

Gender distribution included 14 females (54%), 11 males (42%), and 1 non‐binary participant (4%). The ethnic composition was also varied, with participants identifying as White British, British Chinese, British Indian, Mixed Ethnicity, Black British (Caribbean and African), Asian Indian, and British Pakistani. While White British participants formed the largest group, the inclusion of multiple ethnic backgrounds ensured the intervention addressed issues relevant to underrepresented communities.

Geographically, participants were drawn from across the UK, including North West and South East England, East and West Midlands, Yorkshire, London, and Scotland. This national spread allowed the workshops to reflect regional differences in healthcare access, service provision, and cultural context, crucial for ensuring the intervention is inclusive and applicable across primary care settings in the UK.

### 3.2 Phase 1: Problem Identification

Several challenges emerged across both workshops in response to the three discussion prompts. For Prompt 1, participants identified barriers faced by people with PTSD in the community, which fell into two broad categories: Individual‐level challenges, such as stigma, delayed help‐seeking, social isolation, anxiety, difficulties with daily functioning, and coming to terms with a diagnosis and service‐level barriers, including geographic inequities, long waiting times, poor signposting, delayed or missed diagnoses, and mistrust in healthcare systems or professionals.

Prompt 2 focused on difficulties engaging with a digital app. Individual barriers included digital exclusion, limited motivation, a preference for face‐to‐face support, and difficulties navigating technology. App‐specific issues included concerns about confidentiality and data security, exposure to triggering content, low visibility and promotion of the app, and a lack of personalisation.

Prompt 3 explored clinicians' engagement with the app. Key challenges included limited time, insufficient training or confidence in using digital tools for PTSD, low awareness of the app, concerns around patient safety, and uncertainty about appropriate follow‐up. Supporting File 1 provides a summary of the issues identified across both workshops.

### 3.3 Phase 2 and 3: Solution Generation and Clarification

During Phase 2, participants proposed numerous solutions, which were condensed into 21 unique solutions to enhance the PTSD Hub across both workshops. These included adding new features such as grounding techniques, gamification, and social media integration; improving existing functions by simplifying navigation, allowing more creative expression, creating user sub‐groups, and incorporating diverse modalities; strengthening integration with health services through collaboration, signposting, personalised treatments, and virtual professional meetings; enhancing therapeutic elements like mind‐body therapies and self‐assessment tools; and increasing safety measures such as safety netting and content moderation.

For peer‐led or shared decision‐making features, four solutions were identified: self‐reflection tools, user forums, message boards, and personalised coping strategies.

Regarding clinician engagement, seven solutions focused on involving clinicians in the app's development, boosting awareness and training, and fostering support from patient groups and policymakers.

### 3.4 Phase 4: Ranking

In Phase 4, participants ranked the highest‐rated solutions to improve the PTSD Hub app based on feasibility and appropriateness. Five key themes emerged (see Table 2):

- Signposting to evidence‐based resources, local services, and peer support groups.
- Managing triggers by identifying common triggers and allowing users to hide specific topics or keywords.
- Providing practical advice on coping strategies.
- Enhancing personalisation, including customisation for individual preferences, symptoms, treatments, and daily mood tracking.
- Introducing varied modalities, such as increased audio‐visual content and gamification.

**Table 2:** Solutions devised.

Solutions to improve the PTSD Hub mobile application		
Improvements to the PTSD Hub (Most appropriate, workshop 1)	Improvements to the PTSD Hub (Most feasible, workshop 1)	Improvements to the PTSD Hub (Overall, workshop 2)
1. Signposting to local mental health services and support groups	1. Including more audiovisual modalities; less textual	1. Personalisation and customisation options for symptoms, treatment and progress, user display preferences
2. Including advice on coping strategies	1. Listing common triggers	2. Signpost to other evidence‐based resources
3. Daily mood journaling or symptom tracking features	1. Including advice on coping strategies	3. Hide triggering topics or key words
		3. Gamification
**Solutions to Improve Clinician Engagement**		
**Workshop 1**	**Workshop 2**	
1. Advertise and demonstrate in primary care surgeries and professional meetings	1. Emphasis on the evidence‐based app development process and which elements of the app are evidence‐based	
2. Engage with local rehabilitation services	2. Simplification	
3. Engagement and collaboration with (1) local mental health teams, (2) mental health charities, (3) local peer support groups and (4) primary care clinicians with special interests in mental health/PTSD	3. Signpost to other evidence‐based resources	

For improving clinician engagement, four main themes were identified:

- Emphasising evidence‐based approaches, including transparency in app development and signposting to validated resources.
- Simplifying the app's interface and functionality.
- Raising clinician awareness and knowledge of the app.
- Fostering collaboration with stakeholders such as local rehabilitation teams, mental health services, charities, peer support groups, and clinicians with relevant expertise.

## 4 Discussion

This study aimed to identify ways to optimise an existing digital intervention, the PTSD Hub, with input from individuals with lived experience of PTSD, informal carers, and primary care and mental healthcare clinicians. The findings highlight significant barriers to PTSD care, challenges in digital intervention engagement, and practical strategies to improve the app's usability, personalisation and effectiveness, particularly in peer support, decision‐making, and clinician involvement.

Across phases, clear differences emerged between lived experience contributors and clinicians in how they prioritised improvements to the PTSD Hub. Lived experience participants consistently emphasised usability, emotional safety, and personalised support, prioritising solutions that reduced distress, improved navigation, and enhanced the sense of control when using the app (e.g., options to hide triggering content, practical coping tools, and greater customisation). Their suggestions frequently centred on ensuring the app felt supportive, intuitive, and responsive to fluctuating symptoms and individual needs.

In contrast, clinicians prioritised clinical rigour, risk management, and workflow feasibility. They placed greater emphasis on evidence‐based content, safety protocols, and clear clinical pathways, alongside solutions that supported their ability to engage with the app in practice such as simplified interfaces, training, and integration with existing services. Clinicians were particularly concerned with ensuring the app aligned with professional responsibilities, reduced ambiguity around follow‐up, and supported safe decision‐making.

These differing priorities were complementary: lived experience contributors drove the development of features that promoted autonomy, accessibility, and emotional safety, while clinicians emphasised credibility, accuracy, and integration into routine care. Together, these perspectives shaped a balanced set of recommendations that accommodate both user needs and clinical implementation requirements.

### 4.1 Comparison With Existing Literature

Consistent with the existing literature, participants identified several obstacles in accessing PTSD care, including long NHS waiting times, geographical disparities, stigma, and a lack of trust in healthcare professionals and systems [10]. These factors contribute to PTSD under‐recognition in primary care and delayed treatment, as many patients avoid trauma reminders, a core PTSD symptom, which deters early help‐seeking [1]. Financial constraints and limited awareness of available mental health resources further exacerbate access difficulties [3]. The study also identified barriers to online peer support, such as data security concerns, exposure to emotional triggers, and a preference for face‐to‐face interactions. These findings underscore the necessity for digital interventions to prioritise usability and emotional safety to effectively engage users [15, 16, 17]. Trauma‐sensitive design elements, such as content warnings and app moderation, were also emphasised to foster a safe online environment. These align with evidence that structured support, including self‐monitoring and tailored coping strategies, can bolster user confidence and intervention efficacy [10].

A systematic review assessing engagement with digital mental health interventions more broadly concluded that consulting with those with lived experience during design and development ensures that interventions are appropriately solving problems that users care about [20]; we were able to identify these problems and subsequently work together to co‐produce optimisation elements that address such problems. Existing evidence regarding digital mental health interventions more broadly, often concludes that optimisation techniques such as ensuring well‐integrated and meaningful gamification, and tailoring of programmes to the user improve engagement [20, 21, 22], suggesting that the solutions that arose organically from this workshop focusing specifically on the PTSD Hub and this clinical population were in line with previous evidence in the broader digital mental health intervention literature. Stakeholders recommended several enhancements to improve user engagement and therapeutic impact, including greater personalisation through symptom tracking, customisable interfaces, and gamification, features increasingly recognised for improving adherence and outcomes in mental health apps [10]. These findings extend prior research by demonstrating how stakeholder‐driven refinements can address usability and engagement gaps in digital PTSD interventions.

### 4.2 Strengths and Limitations

A key strength of this study was its co‐production approach, involving people with lived experience of PTSD, carers, and clinicians. This helped ensure the app was designed around real needs and practical experiences. The participant group was diverse, including various ethnic backgrounds and locations across the UK, which supports the relevance of the findings to different populations. Using the NGT allowed us to gather and prioritise a broad range of views effectively. However, this study did not fully meet formal definitions of co‐production, as the app already existed; rather, it represents an optimisation process informed by stakeholder engagement. Recruiting participants mainly through social media may have introduced bias, as not all groups may be equally represented. Also, while this study focused on app development and initial feedback, further testing in real primary care settings is needed to understand its long‐term effects of any changes on user engagement and clinical outcomes.

### 4.3 Reflections on Co‐Production

Co‐production was central to this study, and we actively managed power dynamics through structured methods to ensure each participants opinions are heard and included (Nominal Group Technique) [19], trauma‐informed facilitation, and the involvement of a lived‐experience advisory panel and research partner. These measures aimed to ensure equitable participation and prevent dominance by professional voices. Disagreements did occur, clinicians emphasised clinical rigour, while lived‐experience contributors prioritised usability and safety. Consensus was achieved through ranking and discussion, which elevated features such as trigger‐management controls, simplified navigation, and recovery‐oriented language. Lived‐experience input also shaped personalisation and the inclusion of diverse modalities. This reflexive process highlights that co‐production requires negotiation and transparency.

### 4.4 Implications for Research and Practice

The findings highlight the potential for digital interventions to help fill important gaps in PTSD care access and engagement, especially for patients who face geographic or systemic barriers to accessing services. Primary care plays a key role in managing mental health, and digital interventions like the PTSD Hub could support GPs by providing accessible resources and peer support options.

Online peer support, such as forums and digital communities, offers valuable emotional support and practical coping strategies for people with PTSD. This aligns with existing evidence showing that peer support improves mental health outcomes and can complement clinical care [8, 12, 23]. For the PTSD Hub to be effective in primary care, it will be essential to ensure user privacy, minimise exposure to distressing content, and maintain active moderation of discussions to create a safe environment. Integrating such digital tools into primary care could enhance support for patients while addressing common barriers like limited appointment time and specialist availability. However, successful implementation will depend on clinicians being aware of the app, confident in its use, and supported by clear guidance on how to incorporate it into patient care.

Although primary care is often the first point of contact for individuals with PTSD, recognition and formal diagnosis remain limited [10]. Many GPs face challenges in navigating referral pathways, including uncertainty about appropriate services or delays in specialist access, which may contribute to underdiagnosis and delays in care. Key barriers to clinician use of the PTSD Hub included time pressures, limited PTSD‐specific knowledge, and scepticism about digital interventions. To overcome these, participants recommended raising awareness through primary care networks and professional forums, securing endorsements from respected bodies such as NICE to strengthen credibility, and integrating the app into clinical workflows to facilitate patient monitoring and ease of use.

These strategies reflect wider research showing that clinician familiarity with digital tools, confidence in their benefits, and robust evidence are crucial for uptake [13, 18]. The app's shared decision‐making features were especially valued, as they support patient‐centred care and align with evidence linking shared decision‐making to improved satisfaction, adherence, and mental health outcomes [18, 24].

While this study aligns with existing evidence on the effectiveness of apps and the PTSD treatment model underpinning this intervention, another significant contribution lies in advancing co‐creation in research and treatment development. The integrated and inclusive approach, through the use of a lived experience advisory panel, stakeholder involvement, and structured methods such as NGT [19], demonstrates how collaborative design can enrich both process and outcomes. Our findings show that co‐creation produces highly relevant and actionable data, with implications that extend beyond PTSD or digital interventions. Importantly, people with lived experience were engaged in multiple roles, including research and development, advisory input, and solution‐generation workshops. This model illustrates how embedding practical aims within research and fostering genuine collaboration can inspire new ways of conducting research and developing interventions across diverse contexts.

## Author Contributions

**Natasha Tyler:** conceptualisation, investigation, funding acquisition, writing – original draft, methodology, writing – review and editing, formal analysis, project administration, data curation, supervision. **Sarah Croke:** investigation, methodology, participant recruitment, data curation, formal analysis, writing – original draft. **Chloe‐Nicole Low:** investigation, methodology, participant recruitment, data curation, formal analysis, writing – original draft. **Nicola Cassidy:** conceptualisation, software (mobile application development), investigation, industry partnership, lived experience contribution. **Evgenia Gkintoni:** formal analysis, writing – original draft. **Brian McMillan:** clinical expertise, methodology, recruitment support, study planning, writing – review and editing. **Maria Panagioti:** conceptualisation, investigation, funding acquisition, writing – review and editing, writing – original draft, methodology, supervision, recruitment support, study planning, writing – review and editing.

## Ethics Statement

Ethical approval was granted by the Proportionate University of Manchester Research Ethics Committee (Ref: 2024‐19198‐34681).

## Conflicts of Interest

Nicola Cassidy is the founder of the PTSD Hub mobile application. The other authors declare no conflicts of interest.

## Supporting information

Supplementary_files_PTSD.

## Data Availability

The data that support the findings of this study are available from the corresponding author upon reasonable request.

## References

[1] American Psychiatric Association , Diagnostic and Statistical Manual of Mental Disorders (American Psychiatric Publishing, 2013). 5th ed..

[2] N. Kar , B. Kar , and S. Kar , “Stress, Anxiety, Depression, and Psychosomatic Symptoms in the Times of COVID‐ 19 Pandemic: A Systematic Review and Meta‐Analysis,” World Journal of Clinical Cases 9, no. 27 (2021): 7956–7973.

[3] NHS Digital , Adult Psychiatric Morbidity Survey: Survey of Mental Health and Wellbeing, England, 2014 (NHS Digital, 2016).

[4] B. Pfefferbaum and C. S. North , “Mental Health and the COVID‐19 Pandemic,” New England Journal of Medicine 383, no. 6 (2020): 510–512. 32283003 10.1056/NEJMp2008017

[5] A. Carfì , R. Bernabei , and F. Landi , “Persistent Symptoms in Patients After Acute COVID‐19,” Journal of the American Medical Association 324, no. 6 (2020): 603–605. 32644129 10.1001/jama.2020.12603 PMC7349096

[6] V. Williamson , D. Murphy , A. Phelps , D. Forbes , and N. Greenberg , “Moral Injury: The Effect on Mental Health and Implications for Treatment,” Lancet Psychiatry 8, no. 6 (2021): 453–455. 33743196 10.1016/S2215-0366(21)00113-9

[7] C. W. Hoge , C. A. Castro , S. C. Messer , D. McGurk , D. I. Cotting , and R. L. Koffman , “Combat Duty in Iraq and Afghanistan, Mental Health Problems, and Barriers to Care,” New England Journal of Medicine 351, no. 1 (2004): 13–22. 15229303 10.1056/NEJMoa040603

[8] National Institute for Health and Care Excellence , Post‐Traumatic Stress Disorder. NICE Guideline [NG116] (NICE, 2018). 31211536

[9] J. L. Gradus , O. Shstad , M. Labriola , K. van Ingen , and H. Larsen , “The Relationship Between Screening Positive for Posttraumatic Stress Disorder and the Receipt of Mental Health Care,” Psychiatric Services 69, no. 9 (2018): 1004–1009.

[10] K. H. Seal , “Bringing the War Back Home: Mental Health Disorders Among 103,788 US Veterans Returning From Iraq and Afghanistan Seen at Department of Veterans Affairs Facilities,” Archives of Internal Medicine 167, no. 5 (2007): 476–482. 17353495 10.1001/archinte.167.5.476

[11] R. C. Kessler , “Posttraumatic Stress Disorder in the National Comorbidity Survey,” Archives of General Psychiatry 52, no. 12 (1995): 1048–1060. 7492257 10.1001/archpsyc.1995.03950240066012

[12] National Institute for Health and Care Excellence , Improving Access to Psychological Therapies (IAPT) Programme (NICE, 2021).

[13] K. L. Fortuna , J. A. Naslund , J. M. LaCroix , et al., “Digital Peer Support Mental Health Interventions for People With a Lived Experience of a Serious Mental Illness: Systematic Review,” JMIR Mental Health 7, no. 4 (2020): e16460. 32243256 10.2196/16460 PMC7165313

[14] J. A. Naslund , K. A. Aschbrenner , L. A. Marsch , and S. J. Bartels , “The Future of Mental Health Care: Peer‐To‐Peer Support and Social Media,” Epidemiology and Psychiatric Sciences 25, no. 2 (2016): 113–122. 26744309 10.1017/S2045796015001067 PMC4830464

[15] G. Elwyn , D. Frosch , R. Thomson , et al., “Shared Decision Making: A Model for Clinical Practice,” Journal of General Internal Medicine 27, no. 10 (2012): 1361–1367. 22618581 10.1007/s11606-012-2077-6 PMC3445676

[16] L. A. Shay and J. E. Lafata , “Where Is the Evidence? A Systematic Review of Shared Decision Making and Patient Outcomes,” Medical Decision Making 35, no. 1 (2015): 114–131. 25351843 10.1177/0272989X14551638 PMC4270851

[17] E. M. Boucher and J. S. Raiker , “Engagement and Retention in Digital Mental Health Interventions: A Narrative Review,” BMC Digital Health 2 (2024): 52, 10.1186/s44247-024-00105-9.

[18] J. Lipschitz , C. J. Miller , T. P. Hogan , et al., “Adoption of Mobile Apps for Depression and Anxiety: Cross‐Sectional Survey Study on Patient Interest and Barriers to Engagement,” JMIR Mental Health 6, no. 1 (2019): e11334, 10.2196/11334. 30681968 PMC6367667

[19] J. A. Cantrill , B. Sibbald , and S. Buetow , “The Delphi and Nominal Group Techniques in Health Services Research,” International Journal of Pharmacy Practice 4, no. 2 (1996): 67–74, 10.1111/j.2042-7174.1996.tb00844.x.

[20] D. Z. Q. Gan , L. McGillivray , J. Han , H. Christensen , and M. Torok , “Effect of Engagement With Digital Interventions on Mental Health Outcomes: A Systematic Review and Meta‐Analysis,” Frontiers in Digital Health 3 (2021): 764079, 10.3389/fdgth.2021.764079. 34806079 PMC8599127

[21] J. Torous , J. Nicholas , M. E. Larsen , J. Firth , and H. Christensen , “Clinical Review of User Engagement With Mental Health Smartphone Apps: Evidence, Theory and Improvements,” Evidence Based Mental Health 21, no. 3 (August 2018): 116–119, 10.1136/eb-2018-102891. 29871870 PMC10270395

[22] E. Morton , S. J. Barnes , and E. E. Michalak , “Participatory Digital Health Research: A New Paradigm for mHealth Tool Development,” General Hospital Psychiatry 66 (2020): 67–69, 10.1016/j.genhosppsych.2020.07.005. 32702489 PMC7354771

[23] A. Almuqrin , R. Hammoud , I. Terbagou , S. Tognin , and A. Mechelli , “Smartphone Apps for Mental Health: Systematic Review of the Literature and Five Recommendations for Clinical Translation,” BMJ Open 15, no. 2 (2025): e093932. 10.1136/bmjopen-2024-093932 PMC11815452 39933815

[24] O. Alkhaldi , B. McMillan , N. Maddah , and J. Ainsworth , “Interventions Aimed at Enhancing Health Care Providers' Behavior Toward the Prescription of Mobile Health Apps: Systematic Review,” JMIR mHealth and uHealth 11 (2023): e43561. 36848202 10.2196/43561 PMC10012012

