# Supplementary material for: Optimising the PTSD Hub App Through Co‐Production: Enhancing Digital Support for PTSD Management in Primary Care
Source: Health Expect. 2026 Feb 8;29(1):e70598. doi: 10.1111/hex.70598 (PMC12883668; doi:10.1111/hex.70598)
Supplement: Supplementary file 1 — Supplementary_files_PTSD. [file HEX-29-e70598-s001.docx]

**Supplementary files**

**Supplementary table 1**

**Phase 1: Problem identification**

*** problems identified in workshop 2 only**

| **Prompt 1: What problems do people living with PTSD face in the community?** | | |
| --- | --- | --- |
| **1** | Negative public perceptions and limited understanding of condition | The stigma surrounding PTSD as a mental health condition can lead to misconceptions and misunderstandings among the public. Individuals experiencing PTSD-related symptoms may also fail to recognise that their symptoms relate to PTSD due to stigmatised portrayals of the condition. |
| **2** | Long waiting times for NHS therapy services | Individuals face long waiting lists for a limited number of NHS appointments |
| **3** | Geographical constraints | In-person support services may be far away and difficult to access via public transportation |
| **4** | High costs of accessing services | Individuals may have to resort to seeking private therapy due to long NHS waiting times; the costs associated with travelling to in-person services may be high |
| **5** | Insufficient signposting | A lack of clear guidance may result in individuals being unaware of the available help or the most suitable resources tailored to their specific needs |
| **6** | Delayed help-seeking | Individuals may find it difficult to accept their diagnosis, leading to worsened symptoms and delays in seeking professional help. |
| **7** | Feelings of isolation | Individuals may feel as though they are suffering alone and lack a sense of connection with other individuals with PTSD, which could further prevent individuals from reaching out to their peers about their condition. |
| **8** | Difficulties maintaining relationships | Individuals may face difficulties communicating their feelings with family or friends, which may in turn put a strain on their relationships |
| **9** | Anxiety | Feelings of anxiety around accessing support in the community |
| **10*** | Mistrust of services and professionals |  |
| **11*** | Difficulties accepting a diagnosis | Individuals may find it hard to come to terms with a diagnosis or experience a sense of denial. |
| **12*** | Difficulties with activities of daily living |  |
| **13*** | Delayed diagnoses or misdiagnoses |  |
| **Prompt 2: What problems might people face using the PTSD Hub app (or other digital peer-support solutions)?** | | |
| **1** | Digital exclusion | Inadequate access to the internet or a mobile device may render some individuals unable to utilise the resource, potentially excluding a specific demographic from accessing a key resource |
| **2** | Motivation | Absence of motivation may impede progress or reduce engagement with the necessary interactions on the platform. |
| **3** | Preference in-person support | Some individuals may prefer interacting with a clinician or peers face-to-face, rather than via a digital platform |
| **4** | Difficulty navigating digital technology | Marginalised groups, for example older adults, may encounter challenges in using digital platforms, potentially excluding a specific demographic from accessing a key resource |
| **5*** | Concerns about confidentiality, data privacy and security |  |
| **6*** | Potential triggers | Individuals may find reading about others’ experiences upsetting or triggering, hence avoid using the app. |
| **7*** | Insufficient awareness | Healthcare professionals or individuals with PTSD not being aware of the app would limit its efficacy. |
| **8*** | Insufficient personalisation | Individuals may find that the information given is too generalised, leading to reduced efficacy of the resource in comparison to personalised, in-person services. |
| **Prompt 3: What problems might clinicians face engaging with PTSD Hub?** | | |
| **1** | Time constraints | With limited consultation times, clinicians may not have sufficient time to explore utilising digital interventions with patients when discussing their treatment options. Clinicians also have limited time outside of clinical commitments to familiarise themselves with the resource, hence may not feel confident recommending it. |
| **2** | Lack of expertise/knowledge | Many clinicians lack the training required to understand PTSD management, potentially limiting the efficacy of the resource. |
| **3** | Safety concerns | As there are often so many digital solutions for different conditions, there are concerns around recommending something that is not evidence-based/effective/safe, hence may not feel confident recommending the resource. |
| **4** | Unaware of the resource | Clinicians may not be aware of the availability of the resource |
| **5*** | Difficulties in follow up | Clinicians are not able to track patient progress and interactions on the app, leading to difficulties in follow up and gauging progress. |

**Supplementary table 2**

| **Prompt 1: How could the PTSD Hub be improved?** | | |
| --- | --- | --- |
| **1** | Customisation features | Interactive and personalised app features, such as a daily diary to allow users to track their emotions and symptoms, or elements of motivation such as achievement badges* may encourage progress. |
| **2** | Collaborations with other service providers | Collaborating with other applications could provide users with a wider variety of resources |
| **3** | Clarity and transparency regarding data protection | Ensuring that users are aware that their data will only be used/ retained in line with GDPR regulations may reassure users. |
| **4** | Availability as a website | Having the app available as a website will help individuals who are unable to use an app/ mobile device. |
| **5** | Signposting within the app to evidence-based online or local resources | Providing links or contact details of relevant online resources or local services provides users with more options and can act as important safety netting during the waiting period for specialised support. |
| **6** | Case studies of individuals with lived experience | Experiences shared either as text, video or audio could increase user engagement with the resource. |
| **7** | Varied modalities and features | Incorporating different modalities in the app to convey information e.g. text, audio, video can increase user inclusivity and engagement. |
| **8** | User sub-groups | Including different pages tailored to users in different stages e.g. new vs. experienced users could allow users to engage with features and access information relevant to them. |
| **9** | Grounding techniques | Include information or guidance on grounding techniques or signpost to relevant resources. |
| **10*** | Moderation of content and interactions | Professional moderation of shared content and user interactions on the app may ensure user guidelines are being adhered to. Potential triggers can also be monitored and removed. |
| **11*** | Nudges and notifications | Nudges and notifications may encourage regular app usage. |
| **12*** | Elements of social media | Incorporating elements of popular social media apps, such as short-form mobile videos may encourage user engagement. |
| **13*** | Mind-body therapies | Include information or signpost to resources on holistic, mind-body therapies for PTSD. |
| **14*** | Simplification of features | The app should be simple to use, bearing in mind the effects of mental and physical symptoms of PTSD . Use of text should also be minimised where possible e.g. drop-down menus/ quick selection features can be incorporated in “daily diary” feature. |
| **15*** | Personalised treatment plan | Automatic generation of user progress reports or personalised treatment plans can be shared with friends/ carers/ relatives/ healthcare professions to encourage shared-decision making. |
| **16*** | Safety netting features | Highlight safety netting features such as how to recognise signs of crisis and signposting to urgent help in such an event |
| **17*** | Gamification elements | Features mimicking those of a mobile game could improve user engagement |
| **18*** | Self-assessment tools | Including evidence-based self-assessment tools for PTSD and common co-morbid conditions could complement self-management features |
| **19*** | Virtual meetings with professionals | Offering virtual sessions with mental health professionals can improve user engagement |
| **20*** | Features for creative expressions | Incorporate features that enable users to express themselves in the form of art, music, writing, for example could complement personalisation and customisation of the app |
| **21*** | Indicate the strength of interventions | Incorporating a “traffic-light” system to demonstrate the strength of evidenced-based interventions and strategies gives users a visual guide to aid treatment decisions and self-management |
| **Prompt 2: Which peer-led or shared decision-making strategies for coping with PTSD symptoms could be included in the mobile app?** | | |
| **1** | Self-reflection features | Allowing users to reflect on their progress can be informative and encouraging. |
| **2** | User forums | An online forum or chat group would enable users to interact and share their personal experiences, but care must be taken with regard to forum moderation to enforce user guidelines and regulations. |
| **3** | Message boards | Including a message board for users to share encouraging quotes/ messages can encourage user engagement. |
| **4*** | Coping strategies tailored to individual symptoms and needs | Incorporating personalised coping strategies, integrated into timeframes and a review system can help users prioritise their needs. |
| **Prompt 3: How could we improve clinician engagement with the PTSD Hub?** | | |
| **1** | Include the resource in email communications to clinicians | Promote the app through communication channels with clinicians |
| **2** | Teaching resource for clinicians | A concise teaching guide/ resource aimed at clinicians can increase familiarisation with the app |
| **3** | Include the app on national guidance | e.g. NICE CKS, referral pathways, NHS libraries |
| **4*** | Support from patient, policy makers and commissioning groups | Support from wider patient groups, policy/ commissioning groups (Ii.e. NICE) will demonstrate the app’s evidence-based strategies, efficacy and safety. |
| **5*** | Direct engagement with app developers | Members of the team visiting practices for demonstrations and engagement may increase trust and uptake. |
| **6*** | Involve clinicians in the development process | Including clinicians during app development increases engagement and awareness |
| **7*** | partnerships with healthcare organisations | Collaborating with organisations e.g. RCGP can help promote the app and gather feedback |

*= Solutions identified in workshop 2 only
